# Ethnic Differences in Home-Related Maternal Stress: Muslim and Jewish Mothers

**DOI:** 10.3390/ijerph16224393

**Published:** 2019-11-10

**Authors:** Saadi Diana, Tirosh Emanuel, Agay-Shay Keren, Schnell Izhak

**Affiliations:** 1 Porter School of the Environmental and Earth Sciences, the Faculty of Exact Sciences, Tel Aviv University, Tel Aviv 69978, Israel; 2 Bnei Zion Medical Center, the Rappaport Family Faculty of Medicine, (emeritus), The Technion, Israel Institute of Technology, Haifa 23774, Israel; emi.tirosh@gmail.com; 3 Faculty of Medicine in the Galilee, Bar Ilan University, Safed 5290002, Israel; keren.agay-shay@biu.ac.il or; 4 Department of Geography and Human Environment, Tel Aviv University, Tel Aviv 66978, Israel

**Keywords:** parental stress, maternal stress, heart rate variability as an index of parental stress, socio-economic, demographic, environmental and gender factors associated with maternal stress

## Abstract

Parental stresses are normal responses to raising children. They are affected by stresses parents and children accumulate and bring to their interrelations. Background factors like economic difficulties or the relations between the parents may affect parental stresses as well as demographic and environmental factors like noise and access to urban parks. Most studies on parental stress are based on a verified psychological questionnaire. We suggest using frequency domain heart rate variability index (HRV) to measure parental stress enabling, by thus, the measurement of physiological aspects of stress and risk to health. Parental stress is measured as the difference between HRV accumulated at home while staying with the children and without the husband and HRV measured in the neighborhood while staying without the children and the husband. We use the index to compare differences among Muslim and Jewish mothers in exposure to maternal stress at their homes and to expose the factors that predict differences in maternal stress. We found that Muslim mothers suffer from home-related maternal stress while Jewish mother do not. Number of children and ethnically related environmental aspects predict differences in maternal stress between Muslim and Jewish mothers. Muslims’ lower access to parks stems from lack of home garden and parks in their neighborhoods in the Arab towns but mainly by restrictions on Muslim mothers’ freedom of movement to parks. Despite differences in levels of noise at home and in the status of the mother in the household, these factors did not predict differences in maternal stress. Instead, the study highlights the crucial role of greenery and freedom of movement to parks in moderating home-related maternal stress.

## 1. Introduction

This research aims to investigate maternal stress as reflected by the activity of the autonomic nervous system (ANS) using heart rate variability (HRV) in Jewish and Muslim mothers during their stay with their children at home. HRV is considered a measurement of stress in the short run and as a measurement of risk to health in the longer time. Thus, we test whether Muslim and Jewish mothers are exposed to increased risk to health while staying at home with their children. In addition, we compare the effects of a set of factors including demographic, socio-cultural, women status in their households and environmental ones on maternal stress at home as related to ethnicity.

Stress has been well acknowledged as an important factor related to general and particularly cardiovascular well-being [1]. Furthermore, a substantial albeit inconsistent report of the effects of parental stress on child both physical and mental outcome has been recently reviewed [2].

Parental stress is a normal response of parents to the presence of children in their lives [3,4]. It is rooted in the disparities between expectations to fulfill the parental role and the available resources possessed by parents, and it is affected by the negative stress related to the child and his/her parents [5]. It is widely accepted that parental stress affects both mothers, fathers and children who synergistically affect each other’s coping abilities with their familial lives [6,7,8,9,10]. In these studies, parental stress is determined using psychological questionnaires but not physiological indices as suggested in the present study [11,12]. 

A new review and meta-analysis brings evidences that HRV is a reliable marker of stress [13]. Recent studies have shown that stress affects the ANS and poses risk to health [14,15,16]. HRV measurements are considered important indicators of both physiological resiliency and behavioral flexibility, reflecting the individual’s capacity to adapt effectively to environmental stressors. Previous studies have shown that mental stress is typically associated with increased sympathetic and decreased parasympathetic activity [17,18,19]. These effects on the autonomic tone and balance may consequently result in cardiac rhythm irregularities, as well as disruptions in the immune and hormonal systems [20,21]. Few studies report the effects of disturbed social relations including indices of parental stress on HRV [22,23,24] and only very few studies have tested associations between parental stress and increase in risk to health as being measured by HRV indices [25]. More so, the factors that predict rise in parental stress measured by HRV indices have not been studied.

Parental stress is explained by four main factors including socio-economic, demographic, environmental and women’s status in their households [12]. Previous studies point to the fact that families with low socio-economic status accumulate stress that reduce their parental coping abilities and marital relationship that further affect maternal coping resources with stress [12,26]. Since our main concern is the primary caretaker who spends most of the time with the child as is common in Muslim society in Israel, in the present study we focus on maternal stress.

Studies show that women status in their households and their intra family relations are essential to women’s vulnerability to maternal stress [12,27]. A qualitative study exposed the fact that women’s status in their families was mentioned in open interviews to be a major source of frustration among young Arab women [28]. We also relate to this issue hypothesizing that lower status in the households is associated with increased maternal stress among Israeli mothers.

The protective effects of greenness on health outcomes has been studied extensively (see for a review [29]). Bronfenbrenner [30] stresses the relevance of environmental factors as part of the contextual domain [31]. Among them access to greenery plays an essential role in reducing stresses. Urban parks, including small ones, supply restorative environments to urban dwellers [32,33,34,35,36,37,38] positively affecting humans’ ANS activity [39,40,41,42,43]. These studies focus on the effects of visits to parks on the ANS balance and indicate the direct relations between the extent of green spaces in the city and people’s levels of stress and risk to health [44]. Some of these studies show that even short visits in small urban parks are associated with relaxation [45]. Other studies show that levels of relaxation in green spaces are directly proportional to the amount of greenery in the environment and time spent in green environments [44]. This effect was found valid mainly in lower class neighborhoods where green spaces are relatively scarce [46]. Yet, a whole set of studies argue that even green views at the distance may gain restorative power [47,48]. Unlike the studies that measure the restorative effect of greenery while staying in green places, we evaluate the effect of accessibility to greenery on the stress mothers accumulate in their home environment. We define accessibility in terms of either lack of availability of home gardens or urban parks within walking distance or socio-cultural restrictions on free movement to parks.

A majority of the traditional Muslim women are restricted from visiting public spaces including parks alone [49,50]. A study based on qualitative methods exposed the accumulation of perceived stresses at home and women strive to get more opportunities to relax in parks [51]. Large families, small number of rooms in average houses, restrictions on free movement of many women in outdoor environments, and the fact that husbands frequently commute to work for long hours were found to be associated with the accumulation of stress among Muslim mothers and children [52,53].

Differences in minorities’ responses of the ANS to environmental challenges have been recorded in several studies (e.g., [12,54]). Most studies associate such differences with minority groups’ exposure to discrimination [55,56] or differences in lifestyles [54,57].

We propose that gaining a better understanding of maternal stress as reflected by a physiological measure and possibly identifying stress-moderating factors may contribute to mothers’ well-being. In particular, we address accessibility to green areas as a likely stress- alleviating factor for mothers. We investigate whether these ANS responses, specifically related to a home environment are shared by different ethnic groups. Consequently, we hypothesize that (1) Muslim mothers will endure increased maternal stress in their homes as compared to Jewish mothers. (2) The increased maternal stress may be predicted by a set of demographic, environmental, social and the mother’s status in her household factors.

## 2. Research Method

Seventy-two young, non-smoking mothers aged between 20 and 35 were chosen from two towns of less than 100,000 inhabitants in northern Israel, with 48 of them being Muslim and 24 Jewish. All the women were of middle class and resided in the research area. The research was performed in the neighboring predominantly Jewish town of Afula and the Arab town of Nazareth. They were recruited as a snowball convenient sample. Subject brought other subjects known to them from women in their town. However, significant differences were measured between the Muslim and the Jewish subjects except for their body mass index (BMI). Muslim women who are incorporated into the middle class, work mainly in gendered lower status jobs like teaching, social work etc. Many of them start to join the labor market and to gain higher education during the last decade. Muslim subjects were two years younger on average and they had more children compared to the Jewish mothers [28]. Our sample reflects the ethnic differences that stem from the more traditional norms of the Muslim society as it is further shown in Table 2 in the results section.

This study was approved by the Ethics Committee of the University of Tel Aviv. Before beginning the experiment, a full explanation about the research aim, the experimental procedure, and all measured indices was provided. Informed consent was obtained from all subjects. This study was conducted in accordance with the regulations of the Ethics Committee of the University of Tel Aviv.

After agreement from the subjects, we conducted 12 field campaigns of experiments with six mothers in each campaign. Each session started, at approximately 11:00 a.m. (range between 10:22 to 11:47) in order to account for biological rhythm and climate conditions with the children joining home around 12:00 (the stay at home was with the children all along the experiment). For the purpose of the present report, we selected the data retrieved during visits to two environments in each of the towns respectively: (1) home with the children without the husband; and (2) outdoor residential area without the children and the husband. We identified neighborhood environments that were similar to each other in the two towns in terms of density, types of houses, amount of greenery and low transportation load. Participants stayed half an hour in each environment. Between the research sites, they rested for 15 min in an air-conditioned car.

A specially designed questionnaire was used to record the following independent variables. Background factors: economic status classified by above or below average income; participation in the labor market (yes/no); and education (high school versus higher education). Demographic factors: Number of children (up to two or more than two children at home); house density (up to two or more than two persons per room); and ethnicity. Environmental factors: availability of home garden; a park in a walking distance; and noise at home (above or below 70 dB). Mothers’ status in the household: participation in decision-making (full partnership or less intensive involvement); and freedom to move alone to parks (Yes/No). By this, we adapted Haj-Yahya-Haddad and Schnell’s [28] argument that these two last variables in addition to right for higher education concern young mothers more than any other one in their relations with their husbands. In addition, we related to environmental factors; exposure to noise was retrieved from the monitoring of the visits to the studied sites. Data on thermal load and air pollution that were retrieved from the monitoring process were excluded since their internal variabilities were negligible. Heat loads in the Muslim neighborhood were slightly higher than in the Jewish neighborhood (Mean_Nazareth_ = 19.8; standard deviation (S.D.) = 2.2; Mean_Afula_ = 2.1; S.D. = 2.1; analysis of variance (ANOVA) = 0.1). Levels of CO remained zero in both neighborhoods. In addition, four questions in the questionnaire addressed parks and greenery exposure and access potential.

### 2.1. Devices

The subjects wore a Polar 810i monitor that continuously measured their HRV and automatically calculated the time intervals between subsequent heart beats starting one hour before all sessions. The frequency band includes low frequencies (LF), between 0.04–0.15 Hz and high frequencies (HF), ranging from 0.15–0.4 Hz. The investigator accompanying the sessions wore a Quest Pro device to measure noise levels. We also measured exposure to CO by Pac III, and Kestrel 3000 was used to measure temperature and humidity for climatic variables from which Thermal load was calculated. The accuracy levels of the instruments were secured by applying the calibration methods as instructed by the manufacturers ([45]).

### 2.2. Statistical Analysis

The analysis was conducted in three stages. First, for the 72 participants the mean results of the frequency domain indices of HRV (LF; HF; LF/HF) at home with the children and in the neighborhood without the children were calculated. The exposures measured by HRV indices were normalized by ln functions in order to transform them into close to normal distributions.

Second, we transformed the independent variables into ethnic specific dichotomous variables in order to accommodate for the small sample size. In addition, the dependent variable of the HRV index of stress was analyzed based on the normalized differences in average values of LF/HF at home with the children and without the husband and outdoors in the neighborhood without the children and the husband divided by the value at home.

(LF/HF_Home+children-_LF/HF_neighborhood_)/LF/HF_home+children._

The resulting difference between the indoors with children and the neighborhood without children is used to specifically reflect home-related maternal stress. Each mother has been characterized by one discrete result that represents her average level of maternal stress while staying with the children at home. We further analyzed multiple regressions between the variables that constituted each of the main factors that potentially predict maternal stress. Due to the small sample size we first calculated a separate regression for the independent variables included in each factor predicting maternal stress: Socio-economic; demographic; mothers’ status in their households and environment. These analyses led to a final multiple regression that included only the remaining significant independent variables of the former analyses. We calculated binary correlations between them in order to identify collinearity and removed the independent variables with less predictive value. Finally, the HRV stress index was plotted along the regression line for Muslim and Jewish mothers respectively.

## 3. Results

Comparing the results of HRV while staying with the children at home and those obtained in the neighborhood without the children between Muslim and Jewish mothers reveals a reversed relationship. While the LF/HF ratio appears to be increased at home with the children as compared to the neighborhood without the children among Muslims, an increased ratio is obtained in the neighborhood among Jews. A differential analysis of the specific attributable effect of HF and LF suggests that both HRV frequency bands contributed to these differences. Yet, the parasympathetic effect reflected by the HF plays a more dominant role in Muslim mothers while being in their home with their children (Table 1). 

**Figure 1:**
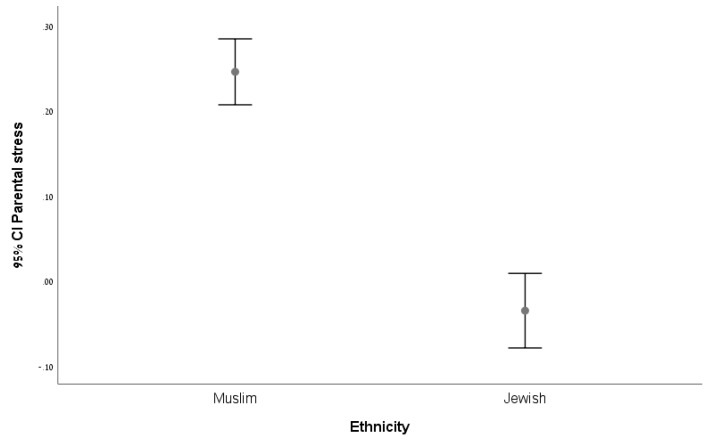
Error bars for Muslim and Jewish home-related maternal stress.

The levels of home-related maternal stress derived from the relative difference between the home and the neighborhood environments in the two ethnic groups, (LF/HF_home+children_-LF/HF_neighborhood_)/Lf/HF_home+children)_ are depicted in Figure 1. This index further supports the difference between the two groups in their ANS activity and specifically in their different activation of the sympathetic and the parasympathetic systems as related to their home environment. All Muslim mothers present increased maternal stress as compared to the majority of Jewish mothers (F = 81.5; Sig. = 0.0001))

The distributions of the dichotomized independent variables for the two ethnic groups are depicted in Table 2. It is evident that Muslim mothers as compared to Jewish mothers run a more traditional lifestyle. They have more children who are born at a younger maternal age, they are less educated, and they live in dense residences. By contrast with the Jewish mothers, about half of the Muslim mothers do not work, they are much less of an equal partner in managing households’ decision-making and they are much more restricted from movement in the urban space, particularly from free movement to urban parks. They live in houses that lack home gardens and in neighborhoods with no parks and their homes are almost twice noisier than Jewish homes.

A multiple regression analyses for both Muslim and Jewish mothers for the variables included in each of the factors that potentially predict maternal stress are presented in Table 3. Socio-economic status only marginally affects maternal stress with education as the only significant variable (R^2^ = 0.17). The demographic factor stands out as the dominant factor predicting 59% of the variability in maternal stress (R^2^ = 0.59), with number of children being the most significant (B = 0.55) while ethnicity although significant appears to be much less related to the stress measured by HRV index (B = 0.22). The mothers’ status in their household and the environmental factor respectively explain between 52% and 55% of the variability in maternal stress. It appears that mothers’ participation in household decision-making does not add to the prediction of maternal stress. Only freedom of movement to parks has a significant effect on maternal stress. Environmentally, despite the high levels of noise in Muslim homes, noise does not add to the prediction of maternal stress. Only the availability of home gardens and parks contribute to the moderation of maternal stress.

Six variables were found significantly related to maternal stress (Table 3). Of those, four were highly inter-correlated with coefficients higher than R = 0.91. Therefore, we related to them as one variable named ethnic-related environment that constitutes freedom of movement to parks, availability of a park and a home garden and ethnicity. Additional regression with the three significant independent variables: number of children; ethnic related environment and mothers’ education, resulted in an increased prediction of maternal stress up to 69% (R^2^ = 0.69) (Table 4).

The main effect of ethnic related environment on maternal stress was supported by answers to four particular questions concerning access to greenery by the subjects. Unlike Jewish mothers, none of the Muslim mothers was allowed to visit parks alone. None of them had a park in their neighborhood while all Jewish mothers had a park in their neighborhood and 86% of the Muslim mothers did not have a garden in their home compared to all Jewish mothers. As a result, two third of the Muslim mothers visited parks less than once a month while most Jewish mothers visited parks at least once a week. A regression analysis between freedom of movement to park and availability of a park in town and a home garden as independent variables and frequency of visits to parks as a dependent variable, reveals that only freedom of movement to parks significantly contributed to the frequency of visits to parks (B = 0.73; Sig. = 0.0001). This explains 47% of the variability in frequency of visits to parks ((R^2^ = 0.47; F = 62.8; Sig. = 0.0001).

**Figure 2:**
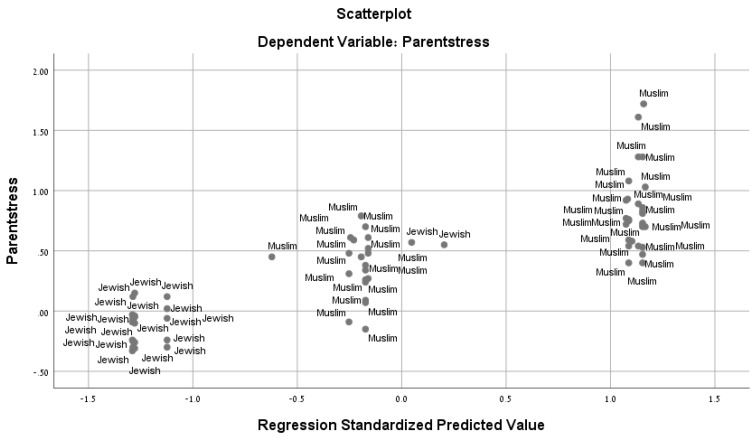
Scatter plot of Muslim and Jewish mothers by maternal stress.

A global Ethnic difference in maternal stress is depicted in Figure 2. The explanatory value of the three variables: number of children, level of education and access to ethnically related environment is highlighted in the scatterplot. It is evident that the two ethnic groups are highly separated along the stress axis following the employment of these three factors. The scatterplot divides the mothers into three groups. On one extreme, are all Jewish mothers (except for two) who are characterized by small number of children and high degree of freedom of movement to parks. On the other extreme are traditional Muslim mothers, whose freedom of movement to park is limited and they tend to have a larger number of children. In the middle are the less-traditional Muslim mothers, whose status in their families is higher but are still limited in their freedom of movement to parks compared to the Jewish mothers. Only two Jewish mothers are located with the Muslim groups along the scatterplot.

## 4. Discussion

This study focused on differences in the degree of home-related maternal stress in Jewish and Muslim mothers and the possible associated risk to health at home. The novelty of this research lies in the application of a physiological index of maternal stress measured by HRV and the specific contribution of staying at home with the children as compared to staying in the neighborhood without the children. It also addresses the effect of social and environmental independent variables that are hypothesized to contribute to home-related maternal stress. We are aware of the fact that homes may stress women in different ways but as the mothers testified in a study of Schnell and Saadi [51], they suffered mainly from maternal stress associated with being rather isolated at home for long hours with their children.

Our first result is that while Jewish mothers do not feel any additional stress while at home with the children, Muslim mothers suffer from increased levels of stress at home with their children. Our second result is that the multiple regression analyses highlight the essential role of number of children and ethnic-related environment in predicting home-related maternal stress. They predict more than two third of the variability in home related maternal stress.

The literature emphasizes a wider set of factors that predict parental stress related to parents, children and environmental and social-background factors [26,31]. We follow Anthony et al. [58] in focusing on parents’ characteristics in explaining parental stress. We take into consideration the synergetic effect of parents on children and vice versa. Stressed parents tend to show less positive affection to their children [59,60], thus intensifying the use of punishments and reliance on corporal punishments [61], leading to a vicious circle of deterioration in family interactions and by thus to deepening maternal stress. Several studies expose the psychological effects of parental stress on children and their parents [6,7,9] They identify deterioration of parents self-images, and relationship [26], as well as parents ability to emotionally support their children [59,60]. 

We follow also Chang et al. (2007) and Bronfenbrenner (2007) in focusing on social and environmental factors that predict maternal stress. As we hypothesized in our second hypothesis in addition to number of children, our study highlight the importance of environmental factors in predicting maternal stress. The results of the present study are also in line with the previously reported increased stress as related to number of children and monotony in life style typical of the Muslim mothers in our study [62]. In our study, the following three ethnic-related environmental factors appeared to affect maternal stress 1. availability of a home garden; 2. a park in the vicinity to the residential area; and 3. freedom of movement to parks. Among them it seems that freedom of movement to parks is the most dominant factor in allowing for frequent visits to parks. However, these factors were found highly correlated as well as significantly related to ethnicity. This collinearity does not allow for a differential weighing of their effects. We interpret this result as a situation in which access to greenery is part of a wider ethnic lifestyle characteristic. Another innovative conclusion is that we find that access to greenery by itself has possibly a restorative effect, while not only visiting or observing green environments but also while the mothers stayed at home with their children.

Ethnicity divides the mothers into three almost exclusive groups: Jewish mothers; traditional Muslim mothers; and in-between modern Muslim mothers and two Jewish mothers. Our results add to the current literature addressing the importance of lifestyle in ethnic related coping with environmental challenges [63,64].

Unexpectedly, noise does not predict maternal stress despite the high exposure of Muslim mothers to levels above the standard of 80 d(B). This contradicts many studies that report strong effect of exposure to noise in cities on HRV [40,65,66]. Our result raises the question whether exposure to high levels of noise from children may differently affect mothers of different ethnicities and or different habitual noise in their environment. Third, mothers’ status in their household as articulated by participation in decision-making did not affect maternal stress. This finding is incongruent with the previously reported maternal stress seemingly related to the perceived mothers’ status in their families and the consequent marital relationships [28]. It is conceivable that the differences in the studied independent variables and the difference in stress measurement modality underlie this difference.

## 5. Limitation

For the purpose of better delineating the factors contributing to the differential stress response as related to home environment and ethnicity a larger and representative sample of Muslim and Jewish participants, both mothers and fathers, is needed. A larger sample will allow a greater number of independent variables in the regressions and will allow isolating more accurately the environmental from the ethnic variables. Such a study would better identify the vulnerabilities related to parental stress in Israel. With the inclusion of participants with a wider range of lifestyle attributes such as freedom of movement and more independent variables including those that focus on the quality of parents’ relations and children characteristics, a more in-depth understanding of the underlying mechanism would be possibly identified. In addition, it is important to create a stratified sample that distinguishes within each ethnic group different levels of access to greenery in order to isolate the effect of access to greenery from the effect of ethnicity. However, even our small sample highlights the categorical differences between Muslim and Jewish mothers in experiencing home-related maternal stress. This highlights the possible importance of access to greenery in the residential environment on moderating maternal stress. Further studies may explore the role of child-care on parental stress by measuring mothers’ levels of HRV at home while the children are in and out of home and by comparing the results to those obtained by standard parental stress questionnaires.

In conclusion, the present study employing ANS balance, using HRV to assess maternal stress response as related to home environment revealed increased stress among Muslim mothers as compared to Jewish mothers. The role of the ethnic-related variables such as number of children and freedom of movement, including accessibility to greenery has been demonstrated.

## 6. Conclusions

Maternal stress is measured here as the difference between HRV at home with the children and without the children in the neighborhood. The main conclusion from the present study is that maternal stress that according to the literature leads to deterioration in the functioning of parents and children may be associated also with increase in risk to health as reflected from their HRV hence their ANS balance. Furthermore, home-related maternal stress may characterize ethnic groups due to their lifestyle as in the case of Muslim mothers in Israel. Environmental factors such as access to greenery articulated by lack of green environments within walking distance and restriction on free movement to more distant green areas play an essential role in predicting maternal stress in addition to the psychological factors. Muslim mothers suffer from unfavorable environmental and social conditions in a way that increases their maternal stress compared to Jewish mothers. At the same token exposure to noise at home did not affect Muslim mothers’ maternal stress.

Highlights: (1) Differences between heart rate variability at home with children and without husband and heart rate variability in the neighborhood without children and husband is considered as an index of parental stress. (2) While Muslim mothers suffer from maternal stress, Jewish mothers do not. (3) Differences between Muslim and Jewish mothers in maternal stress are associated with differences in number of children and access to greenery. (4) Access to greenery is associated with lack of home gardens, parks and mainly women’s freedom of movement to parks.

## Figures and Tables

**Table 1:** Mean frequency domain heart rate variability (HRV) indices with children at home and outdoors without children by ethnicity: Muslim mothers (*n* = 42), Jewish mothers (*n* = 24).

	LnLF/HF			LF_nu			HF_nu		
	Home	Neighbor- Hood	% Diff.	Home	Neighbor- Hood	% Diff.	Home	Neighbor- Hood	% Diff.
Muslims	12.8	8.8	31.2	92.0	88.4	3.9	8.0	11.6	−45.0
Jews	8.2	11.3	−37.8	88.4	91.1	−3.0	11.5	8.9	22.6
% Diff.	35.9	−28.4		3.9	−3.1		−43.7	23.3	
ANOVA Sig.	0.0001	0.0001		0.05	0.001		0.05	0.05	

LnLF/HF=Log normal of frequency domain analysis of the functioning of the autonomic nervous system calculated as the proportion between high and low frequencies. LF_nu=The relative contribution of LF to LF/HF. HF_nu=The relative contribution of HF to LF/HF.

**Table 2:** Distribution of Muslim (*n* = 48) and Jewish mothers (*n* = 24), *n* = (%).

			Muslim	Jewish
Socio-economic Status	Economic status	Low	47.9	0
		Fair through good	52.1	100
	Work	Working	52.1	100
		Not working	47.9	0
	Education	Secondary	64.5	0
		Higher education	35.4	100
Demographic	Residential density	Not crowded	33.3	100
		Crowded	66.6	0
	No of children	Few	39.6	91.7
		Many	60.4	8.3
Woman’s status in Family	Participation in Decision-making	Not involved	66.7	0
		Involved	33.3	100
	Free visit to park	with a company	48.1	0
		On her own	51.9	100
Environment	Garden at home A park in vicinity Noise at home	Yes Yes Average	0 0 88.9	100 100 49.4

Analysis of variance (ANOVA) for differences between Muslim and Jewish subjects for all variables are significant at the level of 0.0001.

**Table 3:** Multiple regression analyses including possible associated stressors as related to maternal stress outcome.

Aspect	Statistics				B	Sig.
	R^2^	F	Sig.			
Socio-economic Status	0.17			Economic status	−0.11	0.8
		7.1	0.002	Work	−0.13	0.8
				Education	−0.38	0.0001
Demographic	0.59	51.0	0.0001	Residential density	0.08	0.92
				No of children	0.60	0.0001
				Ethnicity	0.22	0.0001
Woman’s status in Family	0.54			Participation in Decision-making	0.04	0.3
		41.1	0.0001	Free visit to park	−0.60	0.0001
Environment	0.53	40.9	0.0001	Garden at home A park in vicinity Noise at home	−0.60 −0.60 0.14	0.0001 0.0001 0.51

**Table 4:** A Multidimensional regression between the best predictors of maternal stress and maternal stress.

	B	Sig.	Partial Correlation
No. Children	0.48	0.0001	0.73
Ethnic environment	0.44	0.0001	0.57
Education	0.06	0.46	0.09
